# The Effect of Mixing Entire Male Pigs Prior to Transport to Slaughter on Behaviour, Welfare and Carcass Lesions

**DOI:** 10.1371/journal.pone.0122841

**Published:** 2015-04-01

**Authors:** Nienke van Staaveren, Dayane Lemos Teixeira, Alison Hanlon, Laura Ann Boyle

**Affiliations:** 1 Pig Development Department, Teagasc Animal and Grassland Research and Innovation Centre, Moorepark, Fermoy, Co. Cork, Ireland; 2 School of Veterinary Medicine, University College Dublin, Belfield, Dublin, Ireland; ETH Zurich, SWITZERLAND

## Abstract

Research is needed to validate lesions recorded at meat inspection as indicators of pig welfare on farm. The aims were to determine the influence of mixing pigs on carcass lesions and to establish whether such lesions correlate with pig behaviour and lesions scored on farm. Aggressive and mounting behaviour of pigs in three single sex pens was recorded on Day −5, −2, and −1 relative to slaughter (Day 0). On Day 0 pigs were randomly allocated to 3 treatments (n = 20/group) over 5 replicates: males mixed with females (MF), males mixed with males (MM), and males unmixed (MUM). Aggressive and mounting behaviours were recorded on Day 0 at holding on farm and lairage. Skin/tail lesions were scored according to severity at the farm (Day −1), lairage, and on the carcass (Day 0). Effect of treatment and time on behaviour and lesions were analysed by mixed models. Spearman rank correlations between behaviour and lesion scores and between scores recorded at different stages were determined. In general, MM performed more aggressive behaviour (50.4 ± 10.72) than MUM (20.3 ± 9.55, P < 0.05) and more mounting (30.9 ± 9.99) than MF (11.4 ± 3.76) and MUM (9.8 ± 3.74, P < 0.05). Skin lesion scores increased between farm (Day −1) and lairage (P < 0.001), but this tended to be significant only for MF and MM (P = 0.08). There was no effect of treatment on carcass lesions and no associations were found with fighting/mounting. Mixing entire males prior to slaughter stimulated mounting and aggressive behaviour but did not influence carcass lesion scores. Carcass skin/tail lesions scores were correlated with scores recorded on farm (r_skin_ = 0.21 and r_tail_ = 0.18, P < 0.01) suggesting that information recorded at meat inspection could be used as indicators of pig welfare on farm.

## Introduction

Meat inspection is a promising surveillance tool for pig welfare [1–3]. Being animal based, welfare indicators recorded at meat inspection can provide valuable information about conditions on-farm, during transport, at lairage and pre-slaughter handling [3–5]. Certain injuries and diseases can be detected with high validity and reliability on the carcass and reduce the need for their assessment on-farm [6]. However, research is needed to validate other injuries and lesions recorded on the carcass (i.e. at meat inspection) as welfare indicators. In particular, to differentiate skin lesions arising on-farm during different stages of the production cycle from those arising during transport to the abattoir, at lairage or caused by mechanical damage due to abattoir processes [2]. But also to establish the degree to which lesions recorded on the carcass are correlated to negative behaviours performed by pigs during the production cycle. For example, the relationship between tail lesions scored on the carcass and tail biting behaviour performed by pigs on farm is unknown.

For pigs, mixing with unfamiliar animals is a major source of social stress [7] and typically occurs on the farm prior to transportation to the abattoir, at loading for transport, during lairage or at all three stages [8]. Skin lesions are prevalent welfare outcomes of mixing [9, 10] and skin lesions measured on the carcass are suggested as more sensitive indicators of aggressiveness than those measured on the live pig [11]. Barton Gade and colleagues [12] found that the carcasses of pigs from mixed groups had a higher skin lesion score than those kept in static groups. Fighting increases plasma cortisol levels [8] and higher skin lesion scores are associated with higher plasma cortisol [13]. Skin lesions on the carcass can also be caused by sexual behaviour such as mounting [14] which has negative implications for pig welfare [15]. Entire males are more aggressive and show higher levels of sexual behaviour than females and castrates during the growing/finishing phase [16–19]. Even simple relocation of intact groups of entire male pigs to a novel environment can stimulate mounting behaviour [20]. Hence, the problems associated with mixing pigs prior to slaughter are likely exacerbated in entire male production systems. This may explain why entire males are between 1.3 and 2.5 times more likely to produce carcasses that are downgraded due to lesions than females [7]. Consumer concerns about castration of male pigs have led to an impending voluntary ban on the practice in the EU by 2018 [21]. Hence additional knowledge is required on the impact of routine practices such as mixing prior to slaughter on the behaviour and welfare of pigs in entire male production systems.

In contrast to skin lesions, tail lesions are less likely to be affected by pre-slaughter handling and management. While the stress arising from hierarchal instability caused by re-mixing may indirectly influence the performance of tail biting [22], aggression related to mixing is not directly implicated in the motivation of pigs to tail bite [23, 24].

The main aim of this study was to investigate the effect of mixing entire male pigs prior to transport to slaughter on behaviour and lesions related to pig welfare such as skin and tail lesions scored on the carcass. We hypothesized that mixing would lead to an increase in aggressive and mounting behaviour and as such lead to increased cortisol levels and skin lesions both on the live animal and on the carcass. However, we expected that tail lesion scores on the carcass would be unaffected by mixing. A secondary aim was to establish the relationship between scores of skin and tail lesions on the live animal at different locations (i.e. farm and lairage) and those recorded on the carcass and to determine whether such lesions reflect pig behaviour on-farm and in the pre-slaughter period. The latter will ultimately help to validate meat inspection as a pig welfare surveillance tool.

## Materials and Methods

### Animals and housing

This study was approved by the Teagasc Animal Ethics Committee (TAEC 24/2013). The study was performed on a 1,000 sow integrated commercial farm during the period November 2013 to January 2014. All piglets had their teeth clipped and tails docked and male pigs were not castrated. A wean-to-finish system was used on the farm whereby pigs were grouped according to weight and sex at weaning (approx. 26 days) and after which no mixing of animals occurred until day of slaughter (approx. 5 months). The majority of finisher pigs on the farm were the progeny of Landrace x Large White sows mated to a terminal sire line. However, a proportion of the finisher pigs were Landrace x Large White crosses being the progeny of purebred Landrace sows which were used to produce replacement breeding gilts and represented 20% of the sow herd. Finisher pigs were housed in fully slatted pens (6.4 x 4.6 m) in single sex groups (30.8 ± 6.4 pigs/pen). Pigs had *ad libitum* access to feed from a wet/dry feeder and water was provided by a nipple drinker. Enrichment was provided in the form of a rubber tube hanging from a chain in the pen. Animals were sent to slaughter when they reached a bodyweight of approximately 100–105 kg.

### Experimental design and treatments

Experimental pigs were slaughtered in 5 replicates consisting of 60 pigs each, with all treatments represented on each slaughter day. One week prior to slaughter, three single sex pens (2 entire male and 1 female; hereafter referred to as the ‘home pen’) each containing approximately 30 pigs were selected. A specific number of experimental pigs were randomly selected in each home pen (Table 1) and received individual ear tags. Of these experimental pigs, a number were randomly chosen to act as focal pigs (Table 1) which had received specific ear tags to enable identification. The composition of the groups in the home pens remained unchanged until the day of slaughter. On the day before slaughter all experimental pigs in the three home pens were tattooed with an individual ‘slap mark’ on the left shoulder to enable identification on the slaughterline. On the day of slaughter pigs from the three home pens were allocated to one of three treatment groups (20 pigs/treatment group): 1) male and female pigs mixed prior to transport (MF), 2) male pigs mixed prior to transport (MM), and 3) male pigs unmixed prior to transport for slaughter (MUM). Hence, 20 of the 30 experimental male pigs in the first male home pen (Pen 1) were assigned to the MUM treatment (Table 1). The remaining 10 experimental male pigs in Pen 1 were mixed (MM treatment) with 10 of the experimental male pigs in the second male home pen (Pen 2). The 10 experimental pigs in the female home pen (Pen 3) were automatically assigned to the MF treatment where they were mixed with the remaining 10 experimental male pigs from Pen 2. Six focal pigs were randomly distributed over each treatment with three focal pigs coming from each home pen in the two mixed treatment groups (Table 1). Pigs were held in their treatment groups in three separate holding areas (1.35±0.37 m^2^/pig) in the passageways between buildings on the farm (hereafter referred to as ‘holding’) for approximately 60 minutes before being loaded onto the truck. The order of the groups to be loaded onto the truck was balanced throughout the experiment. Loading and transportation followed routine practice for the preparation of pigs for transport to slaughter. Pigs were loaded onto a three-level truck in their treatment groups at a stocking density in accordance with legislation (minimal space allowance 0.42 m^2^/100 kg pig) and transported for approximately 30 min to the abattoir. After unloading they were held in three lairage pens (0.65±0.03 m^2^/pig) at the slaughterhouse (hereafter referred to as ‘lairage’) for approximately 60 min before being electrically stunned (line speed: 60 pigs/h) prior to exsanguination.

**Table 1:** Distribution of experimental pigs and focal pigs in the home pen on the farm and in the treatment groups after mixing.

	Experimental pigs	Focal pigs	Total pigs
*Home pen (on farm)*			
Pen 1	21 males	9 males	30 males
Pen 2	14 males	6 males	20 males
Pen 3	7 females	3 females	10 females
*Treatment group (holding and lairage)*			
MF	7 males (pen 2)	3 males (pen 2)	10 males (pen 2)
	7 females (pen 3)	3 females (pen 2)	10 females (pen 3)
MM	7 males (pen 1)	3 males (pen 1)	10 males (pen 1)
	7 males (pen 2)	3 males (pen 2)	10 males (pen 2)
MUM	14 males (pen 1)	6 males (pen 1)	20 males (pen 1)

The pen within brackets in the treatment groups indicates from which home pen the pigs originated.

### Behaviour

Pig behaviour was recorded at three stages: 1) in the home pens during the week prior to slaughter; 2) at ‘holding’ on the farm prior to loading and 3) in the lairage pens at the slaughterhouse. Behaviour of all pigs in their home pens was observed continuously on three days before slaughter during four 2-hour periods (Day -5: 15:00–17:00 h; Day -2: 12:00–14:00 h and 15:00–17:00 h; Day -1: 13:00–15:00 h). Three trained observers rotated between the pens every 5 minutes with equal observation times per observer per pen. On the day of slaughter (Day 0) pig behaviour was recorded during holding on the farm and at lairage for approximately 60 minutes at each location.

#### Postures

In the home pens and at lairage, the number of pigs in each posture was recorded by instantaneous scan sampling at 5 min intervals (Table 2). It was not possible to record the postural behaviour of pigs in the holding pens on the farm prior to loading.

**Table 2:** Ethogram of behaviours observed during instantaneous and continuous observations of all pigs in the home pen on the farm or in the treatment group after mixing.

Behaviour	Description
*Posture*	
Lying	Pig lying with eyes open and interacting with something or another pig or with eyes closed and without movement
Sitting	Hindquarters on floor, sitting like a dog (dog-sitting position)
Standing	Pig standing upright on all four feet
*Aggressive*	
Fights	Mutual pushing parallel or perpendicular, ramming or pushing of the opponent with the head, with or without biting in rapidsuccession. Lifting the opponent by pushing the snout under its body [25]. Fights that last longer than 3 seconds are scored as severe fights [26].
Headknocks	Knocking against the head of another pig by a vigorous upward thrust of the head. A succession of 3 head knocks between 2 pigs in retaliation is considered a fight.
*Mounting*	
Mounts	Placing hooves on the back of another pig with or without pelvic movement [27]. Mounts that last longer than 3 seconds are scored as severe mounts.
*Abnormal*	
Tail directed	Tail in the mouth of another pig: ranges from tail being gently manipulated to tail being chewed/bitten
Ear directed	Ear in the mouth of another pig: ranges from ear being gently manipulated to being chewed/bitten
Flank directed	Oral/nasal attention including bites directed towards the flank of another pig

#### Aggressive, mounting and abnormal behaviours

All-occurrence behaviour sampling was used to record the frequency of aggressive (mild/severe fights, head knocks), mounting (mild/severe), and abnormal (tail-, ear-, flank directed) behaviours (Table 2) during five minutes sampling bouts. In interactions where focal pigs were involved, the identity of the pig was recorded and whether it initiated/performed (i.e. actor) or received (i.e. recipient) the behaviour. It was not possible to record the actor/recipient in the case of fights as it was often difficult to determine which pig initiated the fight and there were often more than two pigs involved in a fight.

### Skin lesions on the live animal

All lesions (skin, tail) were assessed by the same observer throughout the study. Skin lesions (i.e. fight-related injuries including scratches and bite marks caused by teeth) on the left side of the body were assessed on all experimental pigs on the farm on Day -1 of slaughter. In addition, skin lesions on the left side of the body were assessed at lairage (Day 0). Due to time constraints only the focal pigs were assessed at lairage. The severity of skin lesions were scored on the back, hind quarter, flank, shoulder, neck and ear (Table 3), following Björklund [28]. Scores from all areas were summed to provide a total skin lesion score, front skin lesion score (sum of ear, neck and shoulder) and back skin lesion score (sum of flank, hindquarter and back) for each pig.

**Table 3:** Skin lesion scoring system.

Score	Description
0	No injuries
1	One small (≤ 2 cm) superficial (pale red) lesion
2	More than one small (≤ 2 cm), superficial (pale red) lesion; or just one red (deeper than score 1) but still superficial lesion
3	One or several large (≥ 2 and ≤ 5 cm) and deep lesions. If deep; only one single lesion, if not so deep; several red lesions
4	One very large (> 5 cm), deep and red lesion. Or many large, deep, red lesions
5	Many very large (> 5 cm), deep and red lesions covering the skin area

Adapted from [28].

### Tail lesions on the live animal

On Day -1 relative to slaughter the tails of all experimental pigs were scored for lesions on a 0–5 scale in the home pens (Table 4). Due to time constraints it was not possible to score tail lesions on any of the animals in the lairage.

**Table 4:** Tail lesion scoring system.

Score	Description
0	No evidence of tail biting
1	Healed or mild lesions
2	Evidence of chewing or puncture wounds, but no evidence of swelling
3	Evidence of chewing or puncture wounds with swelling and signs of possible infection
4	Partial loss of the tail as indicated by an open wound with severe signs of swelling and infection
5	Total loss of the tail as indicated by an open wound at the base of the tail

Adapted from [29, 30].

### Cortisol

Blood samples were collected from all experimental pigs at exsanguination to determine plasma cortisol concentrations. Blood was collected into 10 ml lithium heparin coated tubes (SARSTEDT Ltd., Wexford, Ireland). After collection all samples were centrifuged (2000 x *g*, 4 °C, 10 min) and plasma was separated and stored at -80 °C until analysis by a solid state radioimmunoassay (Cortisol Coat-A-Count, Siemens, Cruinn Diagnostics Ltd., Dublin, Ireland). The intra- and inter-assay coefficients of variation based on controls were 1.5% and 6.5% for low quality controls, 0.9% and 2.5% for medium quality controls, and 0.9% and 1.4% for high quality controls, respectively.

### Skin lesions on the carcass

Carcasses arrived in the chill room (1–4 ^o^C) approximately 25–30 min post stunning, suspended by both hind legs and split in two along the backbone. All carcasses were scored by the same observer who scored the skin and tail lesions on the live animals. Skin lesions on the carcass were scored in a similar manner to those on the live animal. However, areas were adapted to include ear, front (from head to back of the shoulder), middle (from back of the shoulder to the hindquarters), hindquarters and legs (from accessory digit upwards) [5].

### Tail lesions on the carcass

Tail lesions on the carcass of all experimental pigs were scored the same as for the live animals (Table 4).

### Statistical analyses

All statistical procedures were conducted using SAS V9.3 (SAS Inst. Inc., Cary, NC). Residuals of data were checked for normality and transformed where necessary. Statistical differences were reported when P ≤ 0.05 and trends were reported when P values ranged from 0.05 to 0.1. Values are presented as calculated means ± SE, unless stated otherwise.

#### Behavioural data

Behavioural data collected at holding on the farm and at the lairage were analysed using treatment group as the experimental unit (n = 15). Average frequency of behaviours per hour was calculated for treatment groups at holding and at lairage. Data were analysed by PROC MIXED for effects of time, treatment and their interaction with group as repeated subject. A Tukey-Kramer adjustment was used to account for multiple comparisons. Behavioural data that could not be transformed to normality were analysed using the Kruskal-Wallis test separately for holding and lairage (PROC NPAR1WAY). Spearman rank correlations were calculated between the behaviours performed/received by the focal pigs and their lesion scores and cortisol levels (PROC CORR). Specifically correlations were calculated between the sum of behaviours observed in the home pens at the farm (Day -5, -2, -1) and skin and tail lesion scores on the live animals measured on Day -1, skin lesion scores measured at lairage and skin and tail lesion scores on the carcass. Further correlations were calculated between the sum of behaviours observed at holding and lairage and skin lesion scores measured at lairage, skin and tail lesion scores on the carcass and cortisol levels.

#### Live animal and carcass lesions and cortisol

Total skin lesion scores of the live animal were calculated by summing the scores for the separate body parts for all focal pigs. Effects of treatment, time and their interaction on total skin lesion scores were analysed by PROC MIXED using pig as a repeated subject and group as random effect. Effects of treatment on carcass skin lesion scores and cortisol levels were analysed separately for all experimental pigs using group as a random effect (PROC MIXED). Skin lesion scores for the separate body parts (live animal and carcass) were analysed using the PROC GENMOD ordinal model for multinomial data. Spearman rank correlations were calculated between the different lesion scores recorded on the live animal at the farm, at lairage and on the carcass, and between cortisol levels and lesions on the carcass (PROC CORR).

## Results

Two male pigs were considered unfit for transport and were not sent to slaughter. Thus 100 pigs were included in the MF treatment, with 99 pigs in each of the other treatment groups. In total, data were collected for 50 female and 248 male pigs at slaughter.

### Behaviour

#### Postures

Treatment had no significant effect on the postures of pigs in the lairage pens (P > 0.05).

#### Aggressive behaviour

**Fig 1:**
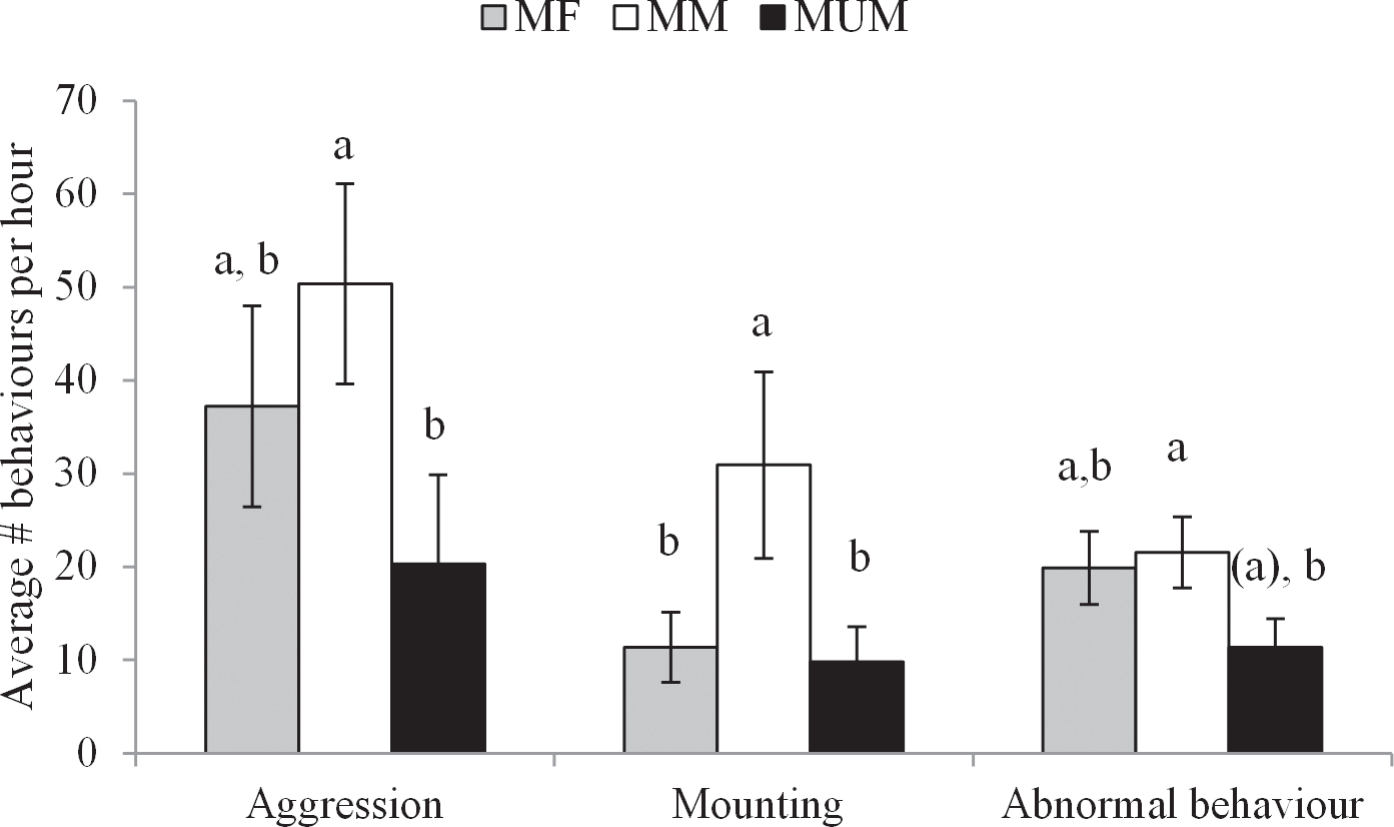
Average number of behaviours per hour at holding on farm and at lairage for each treatment (MF = mixed with females, MM = mixed with males, MUM = unmixed males). Different letters indicate significant differences (P < 0.05), letters within brackets tend to be different from the same letter without brackets (0.05 ≤ P ≤ 0.1).

There were significantly more aggressive behaviours per hour in MM than MUM pigs (P < 0.05), but there were no differences between MF and MUM pigs on the day of slaughter in general (i.e. at holding and lairage, P > 0.05, Fig 1). More aggressive behaviours were observed during holding on the farm than at lairage (56.3 ± 9.61 vs. 19.7 ± 5.42; P < 0.01). There was a tendency for MM pigs to have more severe fights than MF pigs at holding, but no differences were found at lairage (Table 5).

**Table 5:** Average number of behaviours per hour at holding on farm and at lairage for each treatment (MF = mixed with females, MM = mixed with males, MUM = unmixed males).

	MF		MM		MUM		T	Time
	*Holding*	*Lairage*	*Holding*	*Lairage*	*Holding*	*Lairage*		
Aggression	61.7±14.0	17.6±9.15	70.3±15.48	34.4±11.26	36.9±19.44	7.1±1.79	*	**
Fights	19.3±6.30	0.9±0.64	27.0±9.73	1.6±1.07	14.5±8.91	0.3±0.27	ns	***
*Mild*	17.5±5.72	0.9±0.64	18.1±5.86	1.4±1.12	10.7±5.55	0.3±0.27	ns	***
*Severe*	1.8±1.05	0.0±0.00	8.9±4.35	0.2±0.24	3.8±3.51	0.0±0.00	P = 0.08^a^	**
Headknock	42.4±10.92	16.7±8.60	43.3±14.72	32.8±10.40	22.3±10.80	6.8±1.55	*	Ns
Mounting	19.7±6.0	4.7±2.0	55.6±15.0	11.2±1.97	12.4±4.34	7.8±3.94	*	**
*Mild*	15.0±6.09	2.6±0.92	33.4±9.29	5.43±0.92	11.7±3.94	2.7±1.42	*	**
*Severe*	4.7±1.16	2.0±1.23	22.2±10.28	5.7±1.15	0.72±0.45	5.05±4.75	**	*
Abnormal	25.9±6.83	15.1±3.77	18.5±1.67	24.0±6.90	5.3±1.79	16.2±4.28	P = 0.07	ns
Tail	5.3±3.65	0.8±0.56	2.7±1.12	0.7±0.48	1.0±0.56	1.2±0.78	ns	ns
Ear	18.0±4.95	12.5±3.71	14.2±1.59	19.1±5.29	4.3±2.20	11.0±2.91	P = 0.08	ns
Flank	1.8±1.26	0.7±0.44	1.7±0.57	2.0±0.99	0.0±0.00	0.4±0.44	*	ns

Significance levels for treatment (T) and time are given. Treatment by time interactions were not significant (* P < 0.05, ** P < 0.01, *** P < 0.001).

^a^ At holding only.

#### Mounting behaviour

**Fig 2:**
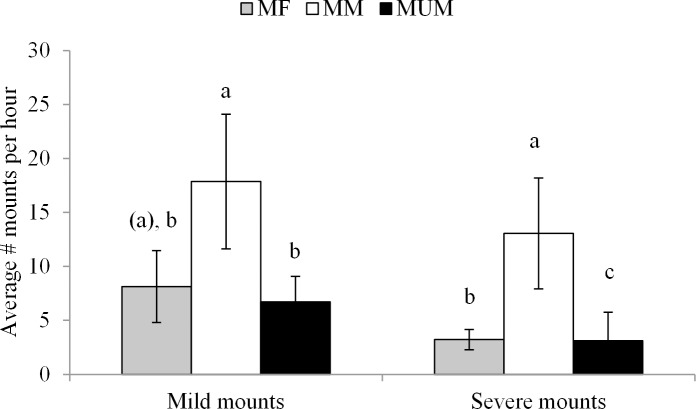
Average number of mounts (mild ≤ 3 sec, severe > 3 sec) per hour measured during holding and lairage for each treatment (MF = mixed with females, MM = mixed with males, MUM = unmixed males). Different letters indicate significant differences (P < 0.05), letters within brackets tend to be different from the same letter without brackets (0.05 ≤ P ≤ 0.1).

Male mixed pigs (MM) performed significantly more mounting behaviour than MF and MUM pigs on the day of slaughter in general (P < 0.05, Fig 1). Similarly, significant effects of treatment were found on the frequency of mild (P < 0.05) and severe mounts (P < 0.01, Table 5). MM pigs showed more mild mounts than MUM pigs (P < 0.05) and tended to show more mild mounts than MF pigs (P = 0.09). MUM pigs showed significantly fewer severe mounts than MM pigs (P < 0.01) and MF pigs (P < 0.05), while MM pigs showed more severe mounts than MF pigs (P < 0.01, Fig 2). Significantly more mounting behaviour was observed at holding on the farm than at lairage (29.3 ± 7.61 vs. 7.9 ± 2.18, P < 0.01).

#### Abnormal behaviours

There was a tendency for an effect of treatment on the expression of abnormal behaviours (P = 0.07). MM pigs tended to perform significantly more abnormal behaviours than MUM pigs on the day of slaughter in general (Fig 1). MM pigs tended to perform more ear directed behaviour (16.9 ± 2.99) than MUM pigs (8.0 ± 2.13, P = 0.08). MM pigs also performed more flank directed behaviour (1.9 ± 0.57) than MUM pigs (0.2 ± 0.24, P < 0.05, Table 5). No significant effect of time was found on abnormal behaviour (P > 0.05, Table 5).

### Skin lesions on the live animal

No significant effect of treatment was found on total skin lesion scores of pigs on farm (i.e. Day -1) and at lairage (Table 6). However, total skin lesion scores were significantly higher at lairage than on the farm (8.1 ± 0.34 vs. 5.7 ± 0.35; P < 0.001). There tended to be an interactive effect between time and treatment on total skin lesion score (P = 0.08). Both MM and MF pigs showed a significant increase from farm (Day -1) to lairage (P < 0.001 and P < 0.01, respectively) while the increase seen in MUM pigs was not significant (P > 0.05). A similar trend was found for the front skin lesion score (sum of ear, neck and shoulder; P = 0.09) which increased significantly in MM pigs (P < 0.01) but not in MF and MUM pigs (P > 0.05), but this trend was not found for the back skin lesion score (sum of flank, hindquarter and back; P > 0.05).

**Table 6:** Average skin lesion scores of pigs at farm (Day -1) and at lairage (Day 0) for each treatment (MF = mixed with females, MM = mixed with males, MUM = unmixed males).

	MF		MM		MUM		T	Time	T*time
	*Farm*	*Lairage*	*Farm*	*Lairage*	*Farm*	*Lairage*			
Total	5.6±0.61	8.2±0.58	5.4±0.54	8.7±0.61	6.0±0.67	7.4±0.59	ns	***	P = 0.08
*Front*	2.9±0.37	3.7±0.31	2.8±0.32	4.2±0.33	2.8±0.36	3.1±0.40	ns	**	P = 0.09
*Back*	2.7±0.38	4.4±0.37	2.6±0.38	4.5±0.39	3.2±0.39	4.3±0.33	ns	***	ns
Ear	0.4±0.14	0.5±0.13	0.2±0.07	0.8±0.13	0.5±0.14	0.4±0.11	ns	*	*
Neck	1.1±0.19	1.3±0.16	1.3±0.23	1.3±0.17	1.1±0.16	1.1±0.17	ns	Ns	ns
Shoulder	1.4±0.21	1.9±0.16	1.4±0.23	2.2±0.21	1.2±0.17	1.6±0.19	ns	***	ns
Flank	1.2±0.20	1.5±0.18	1.2±0.18	1.6±0.18	1.4±0.21	1.4±0.18	ns	P = 0.08	ns
Hindquarter	0.7±0.16	1.6±0.19	0.7±0.18	1.6±0.18	0.9±0.16	1.5±0.15	ns	***	ns
Back	0.8±0.18	1.3±0.18	0.7±0.18	1.3±0.14	0.9±0.19	1.3±0.18	ns	**	ns

Significance levels for treatment (T), time and treatment by time interaction are given (* P <0.05, ** P <0.01, *** P <0.001).

**Fig 3:**
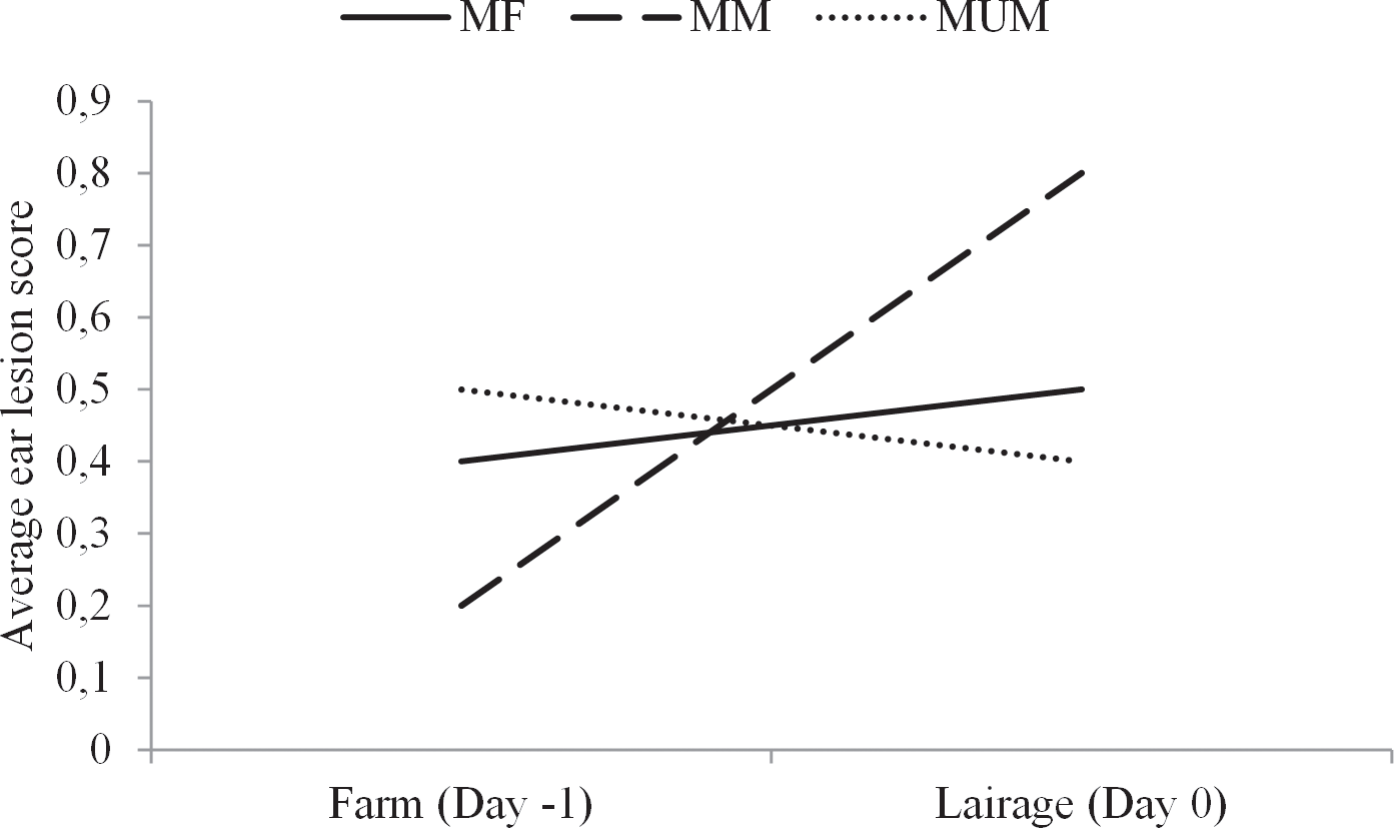
Average ear lesion scores of pigs measured on farm (Day -1) and lairage (Day 0) for each treatment (MF = mixed with females, MM = mixed with males, MUM = unmixed males).

No effect of treatment was found for the skin lesion scores of the separate body parts. Ear lesion scores increased significantly in MM pigs (P < 0.01) but not in MUM and MF pigs between the farm and lairage inspections (P > 0.05, Fig 3). Scores for all body parts, apart from the neck (P > 0.05), increased or tended to increase from farm to lairage (Table 6).

### Cortisol

No significant difference was found between average cortisol levels of MF (410.4 ± 14.96), MM (428.4 ± 18.91) and MUM pigs (442.1 ± 15.58 nmol/L, P > 0.05). No correlations were found between cortisol levels and skin lesion scores of the carcass (P > 0.05). Higher cortisol levels were found in pigs that were involved in more mounting behaviour (i.e. both as actor and recipient, r = 0.26, P < 0.05). Performing more abnormal behaviours (i.e. ear-, tail-, flank-directed behaviour) tended to be associated with higher cortisol levels (r = 0.21, P = 0.08).

### Skin lesions on the carcass

The average scores for the skin and tail lesion scores on the carcass are outlined in Table 7. No significant effect of treatment was found on total skin lesion scores or for skin lesion scores of the separate body parts on the carcass (P > 0.05).

**Table 7:** Average carcass lesion scores of pigs for each treatment (MF = mixed with females, MM = mixed with males, MUM = unmixed).

	Treatment			
	MF	MM	MUM	P
Total	6.1±0.14	6.1±0.15	5.9±0.14	ns
*Front*	2.5±0.08	2.5±0.09	2.4±0.10	ns
*Back*	3.5±0.10	3.7±0.11	3.6±0.10	ns
Ear	0.5±0.06	0.5±0.06	0.5±0.06	ns
Front	2.0±0.05	2.0±0.07	1.9±0.08	ns
Flank	1.9±0.06	2.0±0.07	1.8±0.07	ns
Hindquarter	1.7±0.07	1.7±0.08	1.8±0.06	ns
Tail	1.2±0.06^a^	1.4±0.06^b^	1.5±0.06^b^	**

Significance levels for treatment are given. Different letters in superscript in the same row indicate significant differences (* P < 0.05, ** P < 0.01, *** P < 0.001).

### Tail lesions on the carcass

No severe tail lesion scores (4 or 5) were recorded. Most pigs had mild lesions (score 1: 56.7% and score 2: 36.9%) while 1.7% had moderate tail lesions (score 3). Only 4.7% of the carcasses had no evidence of tail lesions. MF pigs had significantly lower tail lesion scores than MM or MUM pigs (P < 0.01, Table 7).

### Correlations between lesions measured at different stages

Total skin lesion scores measured on farm (Day -1) were positively correlated with total skin lesion scores at lairage (r = 0.45, P < 0.001) and total skin lesion scores measured on the carcass (r = 0.21, P < 0.01). However, only a trend for a correlation was found between the total skin lesion scores measured at lairage and the total skin lesion scores measured on the carcass (r = 0.19, P = 0.07). Tail lesion scores measured on farm were positively correlated with tail lesion scores measured on the carcass (r = 0.18, P < 0.01).

### Correlations between pig behaviour and lesions measured at different stages

Focal pigs that were involved (i.e. as both actor and recipient) in a higher number of fights in holding and lairage had a higher total skin lesion score at lairage (r = 0.23, P < 0.05) but there was no relationship between aggressive behaviour and skin lesion scores measured on the carcass (P > 0.05). Furthermore, there was no relationship between mounting behaviour in holding and lairage and skin lesion scores measured on the live animal (i.e. at lairage) or on the carcass (P > 0.05). Pigs that received more tail directed behaviour in holding and lairage had higher tail lesion scores on the carcass (r = 0.22, P < 0.05).

Pigs that were involved in more fights in the home pens on farm (Day -5, -2 and -1) had a higher total skin lesion score on farm (r = 0.25, P < 0.05) but this did not translate into higher skin lesion scores at lairage or on the carcass (P > 0.05). A higher total skin lesion score on farm was also observed for pigs that were involved in more mounting (r = 0.20, P = 0.05). Pigs that received more mounts tended to have a higher total (r = 0.19, P = 0.07), front (0.20, P = 0.05) and neck lesion score (r = 0.20, P = 0.05). Higher lesion scores for the neck at lairage were observed when pigs received more severe mounts on farm (r = 0.31, P < 0.01). Mounting behaviour observed on the farm was not associated with skin lesion scores measured on the carcass (P > 0.05). Recipients of tail directed behaviour on farm had higher tail lesion scores on farm (r = 0.25, P < 0.05).

## Discussion

In accordance with the literature mixing elicited aggression between pigs [9, 12, 14]. Mixing entire males also resulted in an increase in mounting behaviour and these effects were most pronounced when mixing unfamiliar entire males together compared to mixing males with females. In general, entire males are more aggressive and sexually active than castrates and females [16, 17]; so this is not surprising. However it is the first time that a stimulatory effect of mixing on mounting behaviour has been shown in slaughter pigs. Rydhmer et al. [20] reported similar findings when mixing younger entire male pigs prior to re-locating to the finishing unit. Mixing of either entire males with entire males or entire males with females resulted in an increase in the skin lesion scores measured on the live animals in the lairage compared to their scores measured at the farm on the day prior to slaughter. However, it was only in the male mixed groups that the frequency of aggressive behaviour was higher compared to the unmixed groups. Indeed the difference in aggressive behaviour was mostly related to a higher incidence of head-knocks and such agonistic interactions are less likely to cause damage/injury as less intensive interactions do not always lead to injury [10]. It is possible that considering flank directed behaviour (i.e. flank biting) as an abnormal behaviour [31] was incorrect. In the context of pre-slaughter handling and mixing it is more likely that the behaviour formed part of the pig’s aggressive behavioural strategy and should actually have been recorded as bites. If flank directed bites were included with the other aggressive behaviours then an increase in aggressive behaviour would probably also have been recorded in the treatment where males were mixed with females. This would explain the increase in the skin lesion scores of pigs in this treatment.

Fighting at holding on the farm and in the lairage was positively, though lowly (r = 0.23) correlated with the skin lesion scores of the live animals measured in the lairage but not with skin lesion scores measured on the carcass. Lesion scores are widely used as indicators of post-mixing aggression and slightly higher correlations between fighting and lesion scores (r = 0.40 and r = 0.36) are reported in the literature [9, 10]. The contrast could partially be explained by differences in the way in which lesions were scored in this and the aforementioned studies [9, 10]. Furthermore, in the aforementioned studies the correlations were reported between behaviour and skin lesions on the farm. In the current study the correlation was found for behaviours recorded at holding on the farm and in the lairage and skin lesions scored in the lairage, i.e. after the pigs were transported to the abattoir. Given the numerical though not significant increase in lesion scores of the unmixed pigs between the farm and lairage it is clear that movement/handling and transport alone influence the appearance of skin lesions which could reduce the strength of the correlation between aggressive behaviour and skin lesion scores.

Rydhmer, Zamaratskaia [16] found more scratches on pigs that were being mounted compared to pigs that were not mounted. However, in the current study the increase in mounting behaviour in mixed groups of pigs was not reflected in skin lesions measured on the live animal (i.e. in the lairage). This is in agreement with other studies [15, 20]. Furthermore, we found no relationship between mounting and skin lesion scores measured on the carcass. However, this may have been due to the short latency between the behavioural observations and the scoring of skin lesions on the carcass. Pigs were only observed for 2 hours on the day of slaughter, while in the home pens on the farm in the days preceding slaughter they were observed for 8 hours. This could explain why positive correlations were found between fighting/mounting behaviour observed on farm in the home pens during the days prior to slaughter and skin lesions scored on farm. With the exception of a positive correlation between fighting at holding and lairage and skin lesions measured at lairage, no correlations were found between pig behaviour and skin lesions measured at either lairage or on the carcass. This could suggest that skin lesions are also being influenced by transport and processing on the slaughterline.

We found no effect of either mixing treatment on the skin lesion scores measured on the carcasses. This is in contrast to Rydhmer, Hansson [20] who found a higher proportion of carcasses without lesions and less severe lesions in pigs kept in unmixed groups compared to pigs that were mixed. While D’Eath et al. [32] also found a higher frequency of carcass skin lesions when pigs were mixed, this was only when pigs with highly aggressive temperaments were mixed. Combinations of mixing high and low aggressive temperament pigs, or low aggressive temperament pigs did not affect skin lesion scores or cortisol levels compared to unmixed pigs [32]. This could explain the lack of a difference both in skin lesion scores of the carcass and cortisol levels between mixed and unmixed pigs in our study. However it is also possible that the cleaning and processing of the carcasses on the slaughterline altered the appearance of the skin lesions on the carcass such that the treatment effects seen in the skin lesion scores of the live animals were diminished. Cortisol levels did not differ between treatment groups and were somewhat lower than reported by Chai, Xiong [33] where pigs were slaughtered immediately on arrival at the abattoir. It has been suggested that the effects of loading and unloading are more severe when the time interval between both events is short [33, 34] and a rapid increase in cortisol levels occurs during the first 1.5 hours after loading [35]. Likewise results could be expected for unloading and this could have possibly masked the stress caused by mixing in our study as pigs were slaughtered approximately 1 hour after unloading.

Similar to Harley, More [30] almost all pigs in our study population had detectable tail lesions reflecting the widespread nature of this behavioural vice amongst intensively produced pigs. An effect of treatment was found on tail lesions measured on the carcass where MF pigs had lower tail lesion scores than MM and MUM pigs. We did not expect an effect of treatment as the aggression itself associated with mixing is unlikely to cause tail biting behaviour [23, 24]. Our results seem to indicate that it is not the mixing per se that is a risk for tail lesions as in that case one would expect lower scores in MUM pigs. The reason behind a lower score in MF is most likely due to sex differences (data not shown) as female carcasses are less affected by tail lesions than carcasses of entire males which was reported previously [30]. In addition, the time in between treatment allocation and slaughter was short and consequently the mixing itself could not have had much effect on tail lesions. Possible effects of mixing for a longer duration would have to be considered as industry opinion and anecdotal evidence suggest that mixing can trigger tail biting under commercial conditions [22].

Meat inspection is recommended as a diagnostic tool for pig health and welfare on farm [1–3]. Correlations were found between skin lesions recorded on the carcass, on the farm and at lairage. The correlations were relatively weak, most likely due to the confounding effects of mixing, transport, slaughter and carcass handling on the appearances of the lesions. In spite of this the results suggest that there is still an association between what is visible on the carcass and what is observed on the farm. Tail lesion severity on the carcass also reflected tail lesion severity on the farm. Especially in regards to tail lesions, there are difficulties surrounding scoring on the live animal which make it less accurate then on the carcass. These constraints include dirt on the tail, poor lighting and movement of the animal [6, 36]. The increased visibility make the scoring of tail lesion on the carcass easier than on the live animal and tail lesions are unlikely to be influenced by practices as mixing before slaughter. Therefore, tail lesions recorded on the carcass could be a good welfare indicator to include during meat inspection. This study provides further support for the recording of welfare outcomes at slaughter as a means of monitoring the welfare of pigs on the farm.

## Conclusion

In conclusion, mixing entire males prior to transport to slaughter stimulates mounting and aggressive behaviour, particularly when mixed with other entire males, resulting in an increased risk of skin lesions. However, this damage is not always visible on the carcass and the extent in which slaughterhouse processes change the appearances of skin lesions on the carcass needs further investigation. However, skin and tail lesions measured on the carcass are correlated with lesions measured on the farm suggesting that in spite of these confounding effects of mixing, transport, slaughter and carcass handling, meat inspection can be used to inform farmers about pig health and welfare. These findings have important implications for the development of measurements relating to lesions on the carcass as welfare indicators.
